# Preparation and Characterization of Tilapia Collagen-Thermoplastic Polyurethane Composite Nanofiber Membranes

**DOI:** 10.3390/md20070437

**Published:** 2022-06-30

**Authors:** Sijia Wu, Longhe Yang, Junde Chen

**Affiliations:** 1 Technical Innovation Center for Utilization of Marine Biological Resources, Third Institute of Oceanography, Ministry of Natural Resources, Xiamen 361005, China; gagasaid@163.com (S.W.); longheyang@tio.org.cn (L.Y.); 2 College of Materials, Xiamen University, Xiamen 361005, China

**Keywords:** collagen, Col-TPU composite nanofiber membranes, electrospinning, thermal stability, mechanical properties

## Abstract

Marine collagen is an ideal material for tissue engineering due to its excellent biological properties. However, the limited mechanical properties and poor stability of marine collagen limit its application in tissue engineering. Here, collagen was extracted from the skin of tilapia (*Oreochromis nilotica*). Collagen-thermoplastic polyurethane (Col-TPU) fibrous membranes were prepared using tilapia collagen as a foundational material, and their physicochemical and biocompatibility were investigated. Fourier transform infrared spectroscopy results showed that thermoplastic polyurethane was successfully combined with collagen, and the triple helix structure of collagen was retained. X-ray diffraction and differential scanning calorimetry results showed relatively good compatibility between collagen and TPU.SEM results showed that the average diameter of the composite nanofiber membrane decreased with increasing thermoplastic polyurethane proportion. The mechanical evaluation and thermogravimetric analysis showed that the thermal stability and tensile properties of Col-TPU fibrous membranes were significantly improved with increasing TPU. Cytotoxicity experiments confirmed that fibrous membranes with different ratios of thermoplastic polyurethane content showed no significant toxicity to fibroblasts; Col-TPU fibrous membranes were conducive to the migration and adhesion of cells. Thus, these Col-TPU composite nanofiber membranes might be used as a potential biomaterial in tissue regeneration.

## 1. Introduction

As accidents and diseases lead to tissue damage, natural tissue repair materials are a popular area of research [1]. Collagen has good biocompatibility and can promote cell proliferation and differentiation [2]. What is more, fabricated collagen nanofiber membranes have high-density pores and network structures from micro- to macro-length scales via non-thermal electrospinning. Thus, collagen electrospinning nanofiber membranes enable biomaterials to simulate the extracellular matrix (ECM) environment in vitro [3]. This, in turn, acts as a source of foundation materials for the repair of natural tissue and provides an environment for cell adhesion and proliferation. These collagen-based natural tissue repair materials are frequently used in skin, tendons, blood vessels, as well as nerve and bone regeneration [4,5]. The risk of disease transmission and religious factors have limited traditional collagen such as bovine and pig collagen [6]. Therefore, researchers have focused on searching for marine collagen. Wang et al. separated types I and V collagens from the skin of deep-sea redfish by chromatographic techniques [7]. Chen et al. separated type I collagen from the scales of the lizardfish and maintained the triple-helical structures with no cytotoxicity [8]. Recently, some Japanese scholars extracted collagen from the scales of barramundi (*Lates calcarifer*), which was found to be comparable to that of mammals and showed potential for three-dimensional cell cultivation [9]. Tilapia is a kind of freshwater and saltwater fish promoted by the FAO for aquaculture all around the world, and the production of tilapia is increasing year after year [10]. Marine tilapia collagens have also received significant attention in recent years [11,12,13]. However, marine collagen, without specific shape, structure, or function, cannot be directly applied. Collagen can be compounded with other materials to prepare nanofiber membranes endowed with high-density pores and a network structure with micro- to macro-lengths in scale. Researchers have developed a series of collagen-polymer nanofibrous membranes. Shue et al. fabricated a series of composite fibrous membranes incorporated with fish collagen, nanohydroxyapatite, and poly (lactic-co-glycolic acid) (PLGA) to guide bone regeneration [14]. He et al. prepared collagen-polycaprolactone (PCL) nanofiber membranes, which had diameters of at least 150 nm [15]. However, marine composite fibrous membranes often have limited mechanical properties and poor thermal stability, which hampers their use in tissue repair [16].

Thermoplastic polyurethane (TPU) is an elastomeric polymer with excellent mechanical properties, wear resistance, good elasticity, and toughness [17]. TPU also has good degradability when hydrolyzed, oxidized, or enzymatically degraded in vivo [18]. Routes to degradation of TPU can combine materials in the degradation process to retain CO_2_ and water and modulate changes in pH and chemical stability of the surrounding tissues [19]. Therefore, TPU promotes a stable environment for tissue regeneration; it has good mechanical properties and is environmentally friendly [20]. However, there are few reports of the micromorphology, microstructure, thermal stability, mechanical properties, and biocompatibility of collagen-thermoplastic polyurethane (Col-TPU) composite fiber membranes.

For this reason, the aim of this study was to develop collagen-based nanofibrous membranes that are suitable for the growth of fiber cells and have balanced mechanical properties, good thermal stability, and the potential to become fundamental materials for tissue repair. First, collagen was extracted from tilapia skin. Then, collagen and TPU fundamental materials were used to electrospin a series of Col-TPU nanofiber membranes. The compatibility, micromorphology, microstructure, thermal stability, mechanical properties, and biocompatibility of Col-TPU composite fiber membranes were then studied.

## 2. Results and Discussion

### 2.1. Collagen Structure Identification

**Figure 1:**
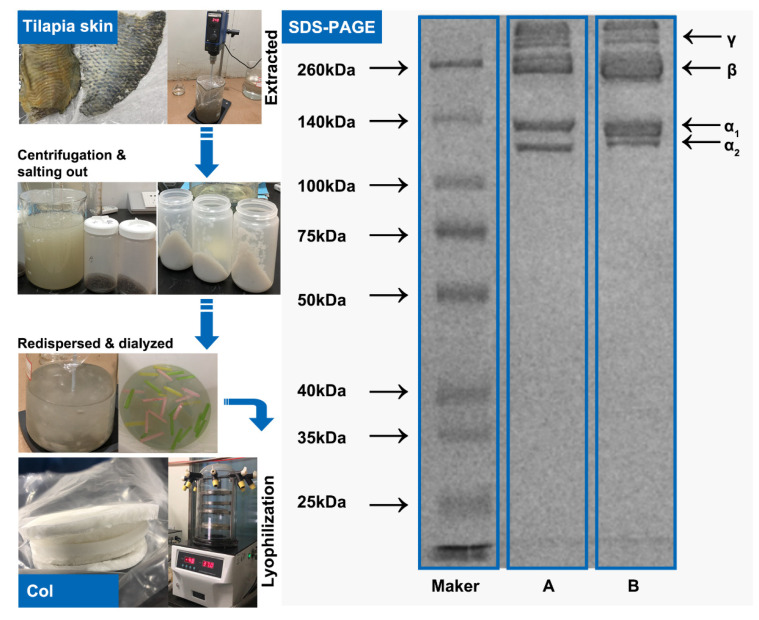
Preparation and characterization of collagen. (**A**) Type I rat tail collagen, (**B**) Tilapia skin collagen.

The sodium dodecyl sulfate-polyacrylamide gel electrophoresis (SDS-PAGE) analysis showed three main bands, as seen in Figure 1. Tilapia skin collagen showed similar electrophoretic patterns to type I rat tail collagen consisting of two different α-chains (α1 and α2), dimeric β-chains, and γ-chains. Tilapia collagen was also consistent with rainbow trout (*Onchorhynchus mykiss*) [21] and black sea gilthead bream (*Sparus aurata*) [22]. Herein, the molecular weight of collagen was analyzed using Quantity One 4.6.0 software (Bio-Rad Laboratories, Hercules, CA, USA). Two bands had molecular weights of 130 and 121 kDa. They were assigned to two α-chains of collagen: α1 and α2 [23]. The two high-molecular-weight components had weights over 200 kDa. These were identified as a β-chain consisting of two α-chains and a γ-chain consisting of three α-chains, respectively [24]. In addition, the ratio of α1 and α2 was calculated with Image J software (VERSION 1.8.0, National Institute of Mental Health, Bethesda, MD, USA); specifically, approximately 2:1 was consistent with the molecular structure of type I collagen [α1]_2_α2, thus indicating that the prepared TSC was type I collagen.

### 2.2. Structural Analysis of Col-TPU Composite Nanofiber Membranes

#### 2.2.1. Scanning Electron Microscope (SEM) Analysis

**Figure 2:**
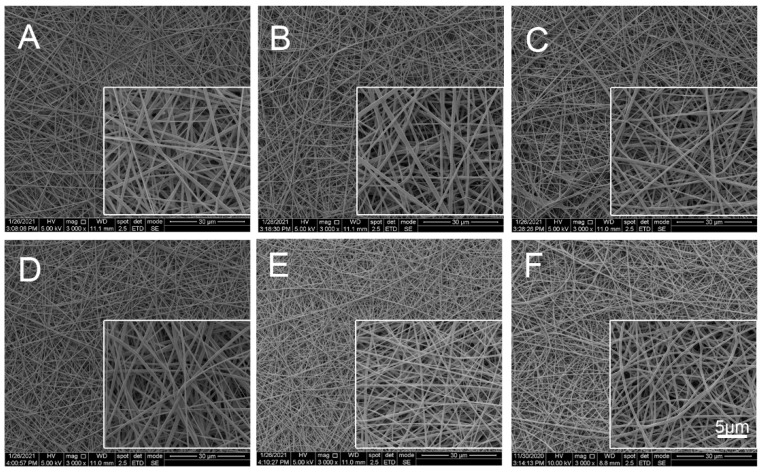
SEM analysis. (**A**) Col100. (**B**) Col95. (**C**) Col90. (**D**) Col80. (**E**) Col60. (**F**) TPU.

SEM (Figure 2) revealed that Col-TPU composite nanofiber membranes (Col95, Col90, Col80, Col60) are uniform and continuous with no beads and better straightness than pure TPU. They range in diameter from 112 nm to 858 nm. The porosity increased with increasing TPU ratios. The average diameters of Col100, Col95, Col90, Col80, and Col60 decreased from 379.96 ± 134.28 nm to 378.40 ± 151.87 nm, 316.80 ± 94.51 nm, 313.80 ± 102.88 nm, and 232.94 ± 87.82 nm, respectively (Table 1). The average diameters of the nanofibers decreased gradually with the increasing compound ratio of TPU. The porosity of all composite nanofiber membranes was higher than 45%, which benefits water and oxygen permeability. The appropriate porosity also facilitates cell migration and cell attachment in the resulting pore structure. 

#### 2.2.2. FTIR

##### Main Characteristic Peak

**Figure 3:**
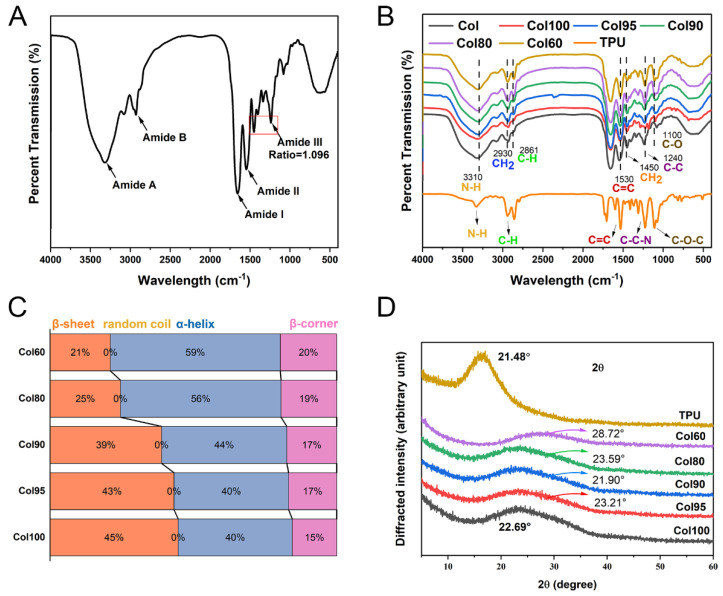
Spectroscopy analysis. (**A**) FTIR of collagen; (**B**) FTIR of Col-TPU composite nanofiber membranes; (**C**) Conformation relative content of amide I band in Col-TPU composite nanofiber membranes; (**D**) XRD of Col-TPU composite nanofiber membranes.

All Col-TPU composite nanofiber membranes have five characteristic peaks that were similar to collagen peaks (Figure 3A,B). The amide A band of collagen was near 3315 cm^−1^, the amide B band was at 2920–2944 cm^−1^, the amide I was at 1625−1690 cm^−1^, the amide II band was at 1500−1600 cm^−1^, and the amide III band was oriented at 1200−1300 cm^−1^. Figure 3B and Table S1 shows that the amide A band of Col100 was oriented at 3311 cm^−1^, and the Col-TPU composite nanofiber membranes were blue-shifted from 3307 cm^−1^ to 3309 cm^−1^ with increasing TPU, which may be caused by the N-H vibration (3310 cm^−1^) coupling effect of the group in TPU. The amide B band reflects the ubiquitinated coupling between the amide A band and the amide II band. Col100 was oriented at 2932 cm^−1^, and Col-TPU composite nanofiber membranes were blue-shifted to 2939 cm^−1^ with increasing TPU. This affected the asymmetric stretching of the C-H group (2942 cm^−1^) in TPU, thus indicating that collagen and TPU were prepared successfully. The amide I and II bands of Col100 were oriented at 1655 cm^−1^ and 1655 cm^−1^. The amide I and II bands of Col-TPU composite nanofiber membranes were ultimately red-shifted to 1554 cm^−1^ and 1534 cm^−1^ with increasing TPU ratios. This may be due to the combination of collagen and TPU, thus showing the characteristic peak of TPU and resulting in a red-shift of the hydroxyl peak. The C=C stretching vibration absorption peak (1532 cm^−1^) of TPU in the Col-TPU composite nanofiber membranes approached the N-H out-of-plane vibration absorption peak at 1537 cm^−1^ in collagen, thus leading to two peaks that expanded into one wide absorption peak on the surface of Col-TPU composite nanofiber membranes [25]. 

The amide III band of Col100 was oriented at 1223 cm^−1^, and the Col-TPU composite nanofiber membranes were blue-shifted to 1230 cm^−1^ upon the addition of TPU, which may affect the vibration of C-C-N in the ethyl carbamate bond (-NHCOO-) in TPU near 1240 cm^−1^. Moreover, the absorption peaks in the region of 1100 cm^−1^ (stretching vibration of C-O-C) in TPU were blue-shifted from 1000 cm^−1^ to 1100 cm^−1^ when the ratio is 60:40 (collagen:TPU). This suggests that TPU combined with collagen to form Col-TPU composite nanofiber membranes during the interfacial interaction between collagen and TPU [26]. The absorption ratio of the amide III band to 1450 cm^−1^ (amide III/A1450) is an important index to determine the integrity of the collagen triple helix structure. When the ratio is <0.5, collagen unwinds the triple helix structure due to denaturation. The absorption ratio of amide III/A1450 in Col100 was 1.114. After adding TPU, and the absorbance ratios of Col-TPU composite nanofiber membranes were 1.131 (Col95), 1.127 (Col90), 1.036 (Col80), and 0.948 (Col60). These data indicate the presence of triple-helical structures of collagen in Col-TPU composite nanofiber membranes [27].

##### Secondary Structure

The secondary structure ratios of Col-TPU composite nanofiber membranes were associated with the amide I band including an α-helix structure, a β-sheet structure, and a β-turn structure. Figure 3C shows that the ratio of β-sheet for collagen decreased from 45% to 21% with increasing TPU ratios, thus suggesting that a more compact structure of the composite nanofiber membrane was formed due to the interactions from larger volumes of side-chain amino acids between collagen and TPU [28]. All Col-TPU composite nanofiber membranes lacked a random collagen structure, suggesting that TPU would not destroy the overall conformation of the secondary structure of collagen. The α-helix ratio of composite nanofiber membranes increased from 40% to 59% with increasing TPU, thus suggesting that the addition of TPU makes sense for the formation of the α-helix. The ratio of the β-turn of Col100 is 14.18%. With increasing TPU ratio, the β-turn of Col-TPU composite nanofiber membranes increased from 15% to 20%. The β-turn mostly transformed into a β-sheet resulting from more -NH groups forming hydrogen bonds, thus indicating that the composite reaction between collagen and TPU is conducive to the formation of an ordered secondary structure leading to a stable collagen structure [29].

#### 2.2.3. XRD

The tertiary structure of the Col-TPU composite nanofiber membranes is shown in Figure 3D. Each Col-TPU composite nanofiber membrane has only one characteristic peak at about 20° in the XRD pattern. This represents the distance between collagen frameworks associated with the diffuse scattering of collagen fibers [30]. These data indicate that the collagen phase does not change upon the addition of TPU. TPU is an amorphous polymer, and there is a wide diffraction peak from 16° to 26° caused by the polyether chain segment in its amorphous structure [31]. 

Figure 3D shows that the wide peak of the Col-TPU composite nanofiber membranes increased to a TPU diffraction peak with an increasing TPU ratio. No obvious characteristic TPU peaks were observed in the pattern. These results indicate that TPU and collagen have high compatibility, and the mixed-membrane matrix can accommodate TPU without affecting its crystal shape. However, 2θ was at 28.72° when the ratio of TPU shifted to almost 40° while maintaining the wide amorphous peak. This suggests that when the ratio of collagen and TPU was lower than 6:4, the tertiary structure of the Col-TPU nanofibrous membrane resulted in a wider diffraction peak. This result was caused by the strong hydrogen bond action between groups of collagen and the secondary amine groups of TPU, which is beneficial in improving the toughness of Col-TPU composite nanofiber membranes [32].

### 2.3. Differential Scanning Calorimetry (DSC)

**Figure 4:**
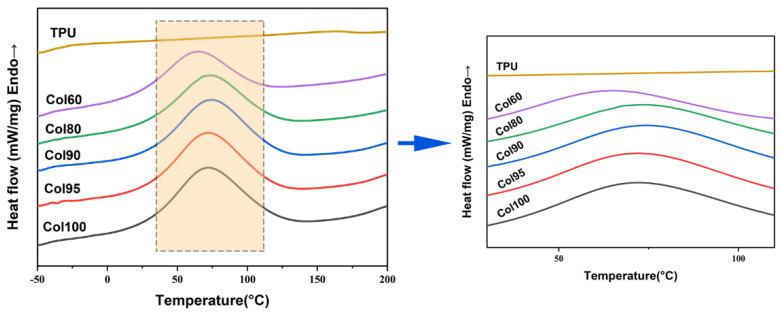
The DSC curves of Col-based composite nanofiber membranes.

The DSC curves of the Col-TPU composite nanofiber membranes are shown in Figure 4. The changes in disappearance of the endothermic peak, the emergence of new peaks, and the change in the enthalpy potentially indicate the components are incompatible [33]. From Figure 4, the endothermic peak of Col100 is 70 °C. There was no obvious peak in TPU because it belongs to the elastomer. Therefore, it does not contain boundaries between soft and hard phases. According to the peaks of collagen-based composites, the addition of TPU in collagen did not generate a new crest, indicating the two had good compatibility. Due to the uniformity of compatibility, the summits of the endothermic peak will be in close proximity to each other if the two phases are compatible [34]. The endothermic peaks of Col95, Col90, and Col80 were 72.1 °C, 74.4 °C, and 73.7 °C, respectively. When the ratio of TPU to composite nanofiber membranes increased to 20, the endothermic peak began to decrease. When the ratio of TPU doubled (60:40), the peak appeared at 65.5 °C. Thus, at the appropriate TPU proportion, the compatibility could be maintained. In addition, the melting enthalpy (ΔH_f_) of Col100, Col95, Col90, Col80, and Col60 was 49.5 J/g, 47.5 J/g, 46.4 J/g, 41.3 J/g, and 31.8 J/g, respectively. The decrease in enthalpy, in association with the changes in the temperature transition, may indicate there is a limited consistency in the boundary of the two phases [35]. 

### 2.4. TGA

**Figure 5:**
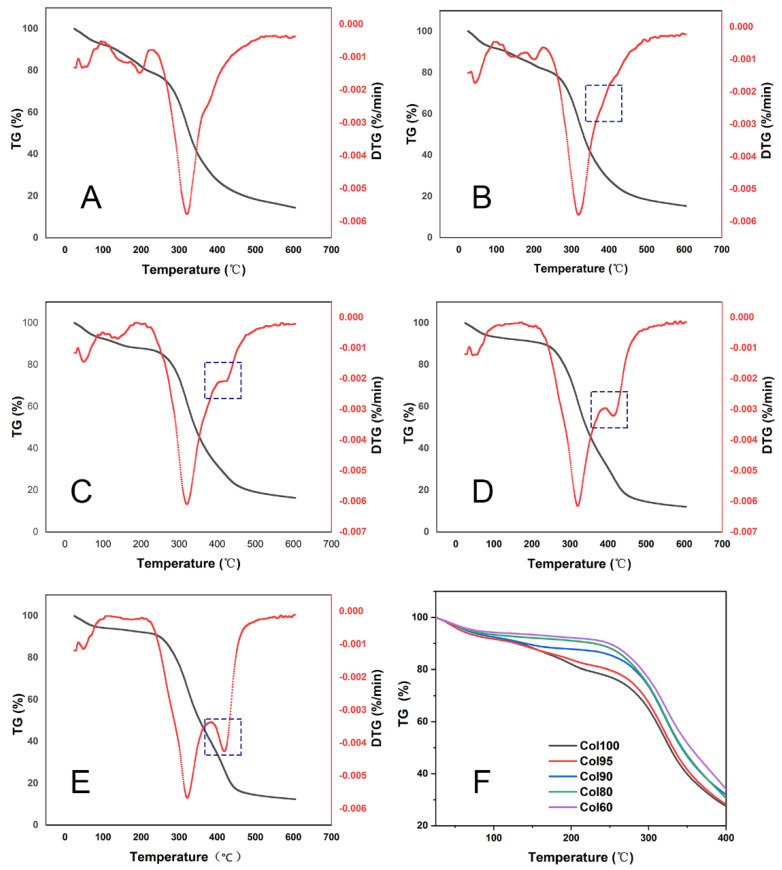
TG-DTA thermogravimetric curve. (**A**) Col100; (B) Col95; (**C**) Col90; (**D**) Col80; (**E**) Col60; (**F**) Local enlarged view of TG curve.

The thermal stability of Col-TPU nanofibrous membranes was evaluated by thermogravimetric analysis (TGA). The thermogain-loss trend of all samples can be divided into two stages (Figure 5A–F). In stage one, the temperature leading to a 5% weight reduction is caused by the evaporation of free water in the sample defined as T5% (Table S2). The T5% of the Col-TPU composite nanofiber membranes with different ratios are roughly the same. When the ratio of TPU increased to 40, the weight loss temperature of Col-TPU nanofibrous membranes increased from 65.0 °C to 75.7 °C, which implied enhanced water retention of nanofibrous membranes. Col100 and Col-TPU composite nanofiber membranes have weight loss caused by thermal decomposition in stage two. The temperature of the maximum decomposition rate (Tp) was used to characterize this thermal decomposition temperature [36]. The weight-loss curves of all Col-TPU composite nanofiber membranes were higher than those of Col100. Table S2 shows that the Tp in Col100 is at 314 °C. With increasing TPU, the Tp of Col-TPU composite nanofiber membranes gradually reached 314 °C, 320.5 °C, 320.7 °C, and 321.8 °C, respectively. These data indicate that the thermal stability of Col-TPU composite nanofiber membranes improved versus collagen with increasing TPU. We also investigated the weight-loss stage when the weight dropped by half (with the decomposition temperature defined as T_50%_). This was a good metric of thermal stability. When the ratio of TPU increased, the T_50%_ of Col100 changed from 327.0 °C to 331.0 °C, 342.3 °C, 343.0 °C, and 353.7 °C. These further indicated that the addition of TPU led to better heat resistance with improved decomposition temperatures. At the same decomposition temperature, the residual Col-TPU composite nanofiber membranes were 5% higher than that in Col100 after heating. The Col-TPU composite nanofiber membranes had less decomposition at high temperatures. These data indicate that TPU might slow the decomposition of the collagen matrix and prevent external diffusion and release, thus improving the thermal stability of Col-TPU composite nanofiber membranes.

### 2.5. Water Contact Angle (WCA)

**Figure 6:**
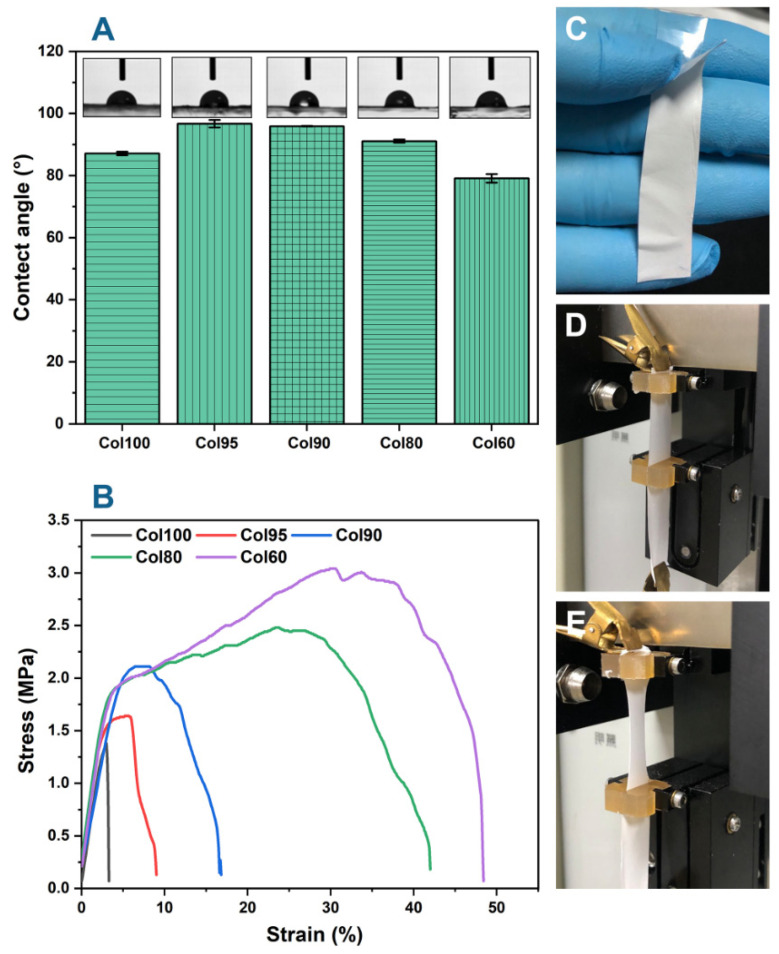
Properties of Col based composite nanofiber membranes. (**A**) WCA; (**B**) Stress–stain curve; (**C**) Prepared Col-TPU composite nanofiber membrane; (**D**) Before tensile test; (**E**) After tensile test.

The hydrophilicity of Col-TPU composite nanofiber membranes was assessed with the WCA. Figure 6A shows that the WCA of Col100 was 87.1 *±* 0.6°, thus indicating that collagen is hydrophilic [37]. When the ratio of TPU increased, the WCA of Col-TPU composite nanofiber membranes increased to 96.7 *±* 0.0°, 95.5 *±* 0.1, and 91.1 *±* 0.5°. The diameter of fibers, the surface roughness, and the pore structure of the membrane affect the hydrophilicity of the material [14]. TPU decreased the diameters of the nanofiber membrane so that the surface of the membranes had more visible pores. This further weakened the barrier properties of the composite material, making it easy for water to penetrate and leading to a lower water contact angle [38]. Moreover, Col-TPU composite nanofiber membranes are hydrophobic, which may be caused by electrospinning disrupting the hydrophilic balance and producing a higher WCA. When the ratio of TPU increased to 40, the WCA of the Col-TPU composite nanofiber membrane (Col60) was 79.1 *±* 1.4°, thus indicating that the wetting behavior of Col-TPU composite nanofiber membranes gradually changed from hydrophobic to hydrophilic. These data indicate that the hydrophilic groups on the TPU molecular chain gap and collagen microfiber were transferred to the side of the low-moisture section after absorbing water at the high-moisture section when TPU reached a certain ratio [39]; this, in turn, increased the hydrophilic groups in the Col-TPU composite nanofiber membranes and greatly increased the water absorption performance of the composite nanofiber membranes. These observations were consistent with the FTIR results. 

Appropriate hydrophilicity has great significance for biomaterials, and improved surface hydrophilicity is expected to promote cell adhesion and proliferation [40]. Thus, the proper addition of TPU can lead to stable hydrophilicity of Col-TPU composite nanofiber membranes. This, in turn plays an important role in improving the adhesion and growth of cells on the surface of nanofibers.

### 2.6. Mechanical Properties

The mechanical properties of Col-TPU composite nanofiber membranes were evaluated as shown in Figure 6B and Table 2. The breaking strength reflects the anti-aging ability of the material [41]. The breaking strength of Col100 is 36.21 cN, which is lower than all Col-TPU composite nanofiber membranes. With an increase in the TPU ratio, the breaking strength gradually increases from 43.26 cN (Col95) to 56.28 cN (Col60), thus indicating that the Col-TPU composite nanofiber membranes had better wear resistance and mechanical durability. Elongation at breaking reflects the toughness and elasticity of the composites’ mechanical properties. The elongation at the breaking of Col100 is 3.20%, which has tensile mechanical properties caused by the relaxation process immediately after fiber formation. However, during the relaxation process, the poor deformation ability of the collagen molecular chain and the loss of molecular orientation generate poor tensile properties. 

TPU is an elastic body with excellent elasticity and wear resistance. It is very soft and flexible with a low modulus. After adding TPU, the values for elongation at break of Col95, Col90, Col80, and Col60 were 8.90%, 16.50%, 41.90%, and 48.30%, respectively. The elongation at break of Col-TPU composite nanofiber membranes increased significantly with increasing TPU content. Tensile strength is commonly used to describe the external force that the composite material can bear, which in turn depends on the maximum external force for each fiber in the unit area; Col100 was 1.40 MPa. As TPU increased gradually, the tensile strength of Col95, Col90, Col80, and Col60 increased to 1.64 MPa, 2.12 MPa, 2.49 MPa, and 3.05 MPa, respectively. The tensile strengths of Col90, Col80, and Col60 were similar to the tensile strength of human tissues [42]. For instance, the native blood vessel structures in the human body, such as the left internal mammary artery (4.1–4.3 MPa), saphenous vein (1 MPa), and femoral artery (1–2 MPa), limit the burst strength to prevent rupture due to variation in blood pressure [43]. Similarly, stiffness of ECM in vivo was verified in the approximate range of 0.1 kPa (brain tissues) to 100 GPa (bone tissues) [38].

Ideal tissue repair materials are expected to have sufficient long-term mechanical properties to support tissue growth—especially scaffold materials such as tissue-engineered cartilage, bone, muscle legs, and ligaments [44]. Furthermore, the excellent elastic properties can withstand repeated dynamic loads and maintain structural stability to simulate human tissues; these properties are needed for tissue engineering applications in the heart, skin, blood vessels, and cartilage [45]. The ratio of TPU in Col-TPU composite nanofiber membranes significantly affected the mechanical properties. Col-TPU composite nanofiber membranes give the material better flexibility and can lead to close contact with the surrounding tissues after implanting the chosen tissue repair material [39]. Col-TPU composite nanofiber membranes have high strength, good anti-aging ability, and suitable flexibility in comprehensive mechanical properties. As such, Col-TPU composite nanofiber membranes can theoretically contribute to a stable environment for tissue regeneration.

### 2.7. Cytocompatibility

#### 2.7.1. Cell Proliferation and Cytotoxicity 

**Figure 7:**
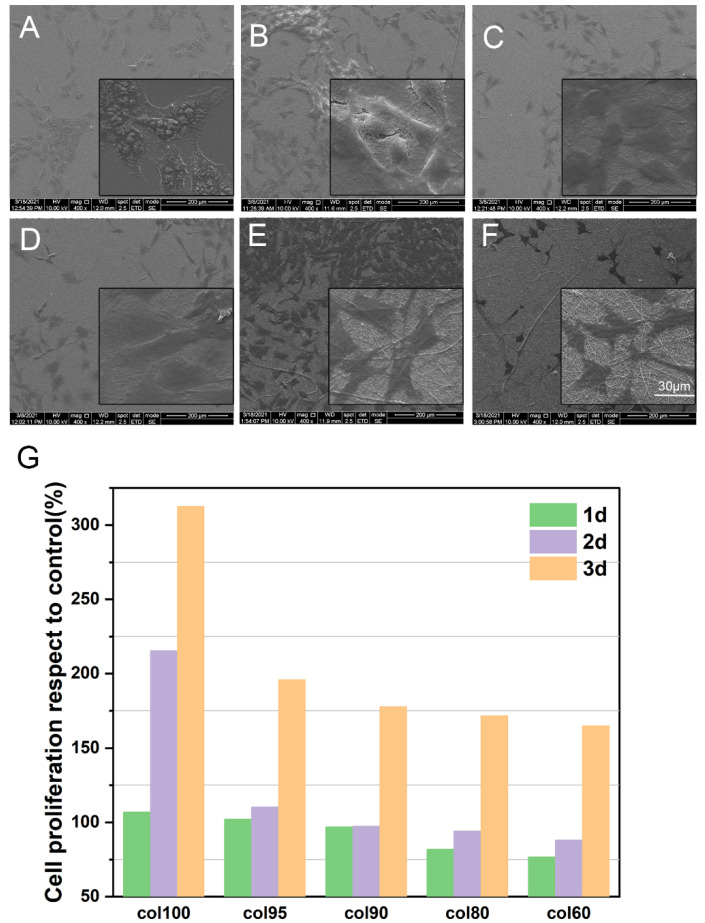
Growth of MC3T3-El cells on collagen-based composite nanofiber membranes. (**A**) Control; (**B**) Col100; (**C**) Col95; (**D**) Col90; (**E**) Col80; (**F**) Col60; (**G**) Cell proliferation rate.

To evaluate the biocompatibility of Col-based composite nanofiber membranes, a CCK-8 assay was used to investigate the proliferation of MC3T3-E1 cells on Col100 and Col-TPU composite nanofiber membranes for 1, 2, and 3 days (Tables S3 and S4 as well as Figure 7G). After culturing for 3 days, all samples showed a significant difference compared to the control group with higher absorbance. The proliferation rate of all samples was higher than 164% after 3 days of culture, which was higher than that of polycaprolactone/poly composite nanofiber membranes (approximately 111%), polycaprolactone/poly/hydroxyapatite composite nanofiber membranes (approximately 112%), and silk fibroin nanofiber membranes (ranged from 83% to 90%) [46,47]. The cytotoxicity assay is also an important index reflecting the biocompatibility of fabricated materials. Specifically, the cytotoxicity of composite nanofiber membranes was grade 0, according to the ISO standard (ISO10993.12-2004). The presence of collagen on composite nanofiber membrane surfaces improved the tendency of cells to adhere to the scaffolds [48]. Col-TPU composite nanofiber membranes support cell growth.

#### 2.7.2. Cell Morphology

The morphology of MC3T3-E1 cells cultured on Col100 and Col-TPU composite nanofiber membrane surfaces was preliminarily evaluated by SEM analysis; the cell morphology can be observed in Figure 7A–F. After 3 days of culture, the cells grew well on Col00 and Col-TPU composite nanofiber membranes and showed a natural spindle shape. Similar changes in morphology were found in Liu’s research [49]. These Col-TPU composite nanofiber membranes were well distributed with increased pseudopodia and spreading area. Compared to the control group (Figure 7A), cells adhered to fibers well, thus indicating that Col-based composite nanofiber membranes can support cell adhesion and diffusion.

#### 2.7.3. Cell Adhesion

**Figure 8:**
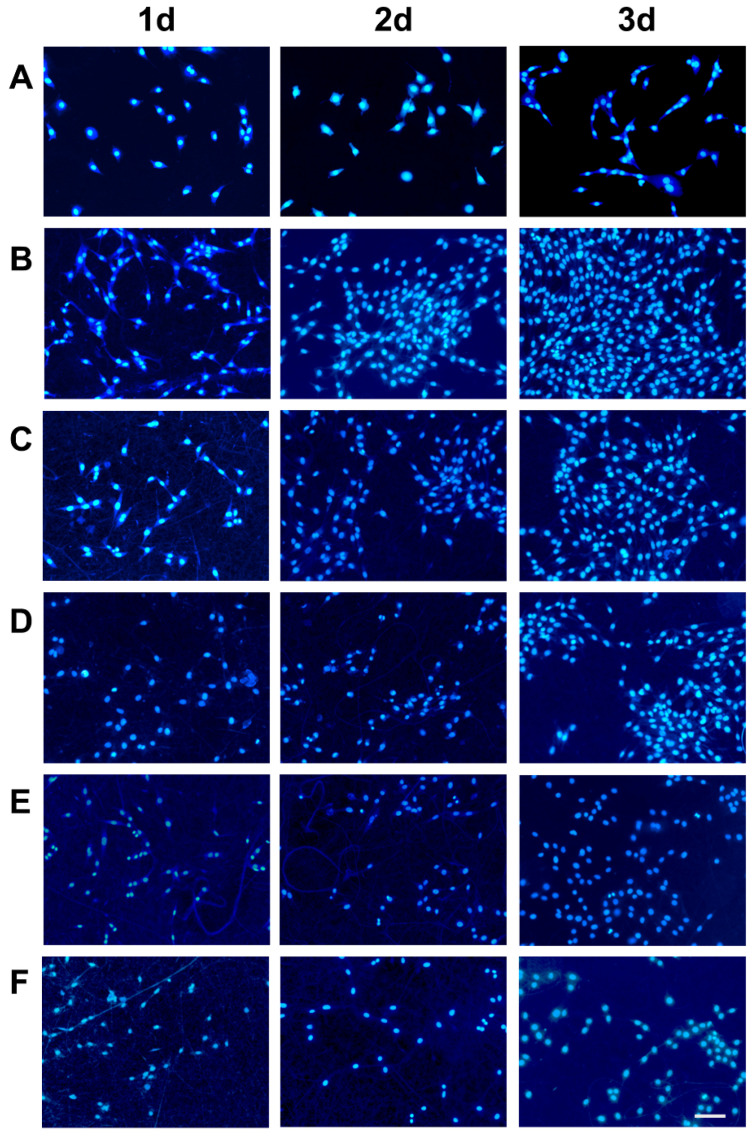
Positive fluorescence microscope scan of MC3T3-El cells on Col-based composite nanofiber membranes; scale bar is 150 μm. (**A**) Control; (**B**) Col100; (**C**) Col95; (**D**) Iol90; (**E**) Col80; (**F**) Col60.

Figure 8 shows that MC3T3-E1 fibroblasts adhered and diffused evenly along the fibers inside and on the surface of the Col100 and Col-TPU composite nanofiber membranes. The cells adhered firmly with a normal and round shape, thus presenting a dense amount along the length of the fiber edges. The number of high-density cells was observed over time. There were more cells at 3 days than at 1 day, which is consistent with the CCK-8 cell proliferation assay. Few apoptotic nuclei were observed on Col100 membranes, which indicated that collagen may affect intracellular signaling and cellular responses [50]. As the collagen ratio decreased, fewer cells adhered to Col-TPU composite nanofiber membranes.

**Figure 9:**
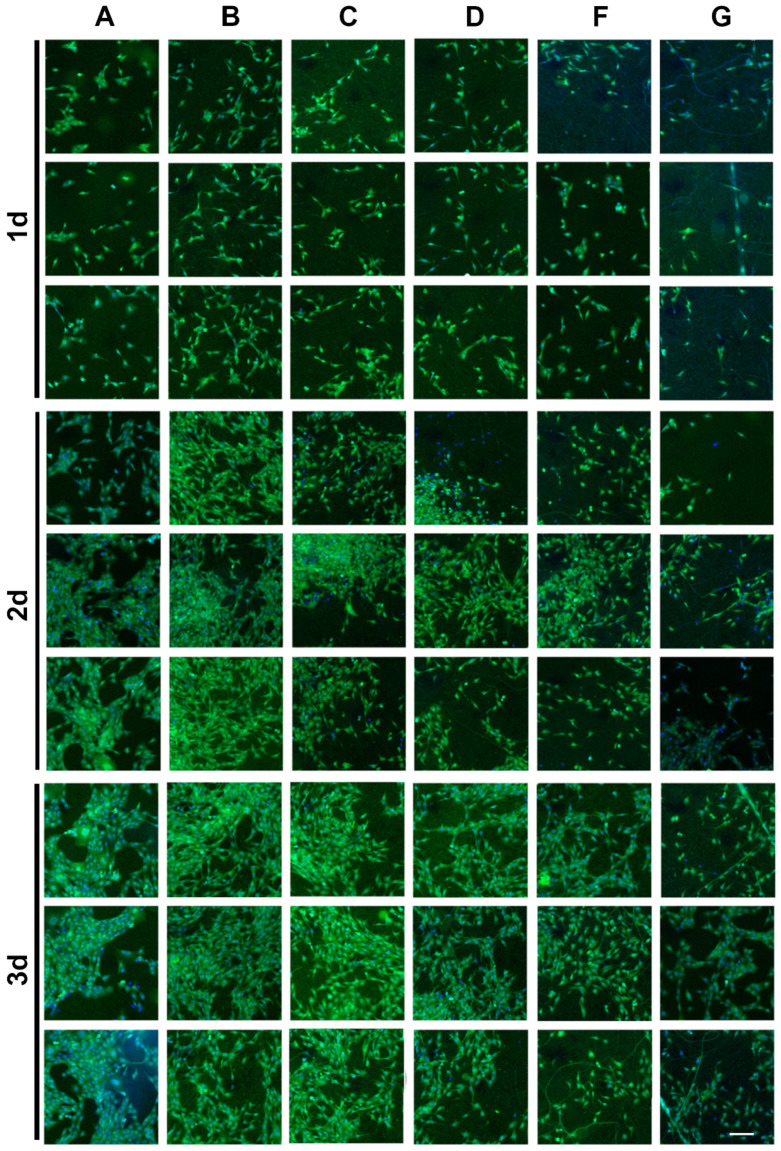
HCA scan of MC3T3-E1 cells on control and samples (green: cytoplasm; blue: nucleus); scale bar is 150 μm. (**A**) Control; (**B**) Col100; (**C**) Col95; (**D**) Col90; (**F**) Col80; (**G**) Col60.

Figure 9 and Figure S2 show similar trends. After 3 days, there were many viable cells filled with MC3E3-E1 fibroblasts on the material’s surface. Col100 was significantly better than the Col-TPU composite nanofiber membranes. The number of adherents decreased with reduced collagen proportion in the Col-TPU composite nanofiber membranes. Identifying the appropriate ratios between collagen and TPU is key to the success of the material. As a result, processed tissue engineering materials can be developed with the desired properties and biocompatibility.

Overall, the Col-TPU composite nanofiber membranes promoted migration and adhesion of MC3T3-E1 cells on the surface of the materials and also supported the proliferation of MC3T3-E1 cells on the surface. This result was consistent with the conclusions of similar experiments [51], thus indicating that the Col-TPU composite nanofiber membranes have good biocompatibility.

## 3. Materials and Methods

### 3.1. Materials

Tilapia (*Oreochromis nilotica*) skin was obtained from Beihai Quality Aquatic Products Co., Ltd. (Beihai, China). Type I collagen from rat tail and protein markers (26634) were purchased from Thermo Fisher Scientific (St. Louis, MO, USA). TPU was purchased from Dongguan Jiayang New Material Technology Co., Ltd (Dongguan, China). The 1,1,1,3,3,3-hexafluoro-2-propanol (HFIP) was from Sigma-Aldrich (Shanghai, China) Trading Co., Ltd. (Shanghai, China). The separating gel buffer (pH = 8.8), stacking gel buffer (pH = 6.8), sodium dodecyl sulfate (SDS), and loading buffer (5×, with DTT) were purchased from Beijing Solarbio Science & Technology Co., Ltd. (Beijing, China). Coomassie Brilliant Blue R-250 and N,N,N′,N′-tetramethylethylenediamine (TEMED) were obtained from Bio-Rad Laboratories (Hercules, CA, USA). Coomassie Brilliant Blue (CBB, R-250) was purchased from Sinopharm Chemical Reagent Co. Ltd. (Shanghai, China). Phosphate buffer solution (PBS, pH = 7.4) was purchased from Wuhan Servicebio Technology (Wuhan, China) Co. Ltd., and potassium bromide (KBr, spectral pure) powder was purchased from PIKE (Mount Airy, NC, USA). Mouse embryo osteoblast precursor (MC3T3-E1) cells (Cat No. CBP60946) were provided by Cobioer (Nanjing, China). The pression vector (LV-GFP) was synthesized by Amer Genomics Biotechnology Co., Ltd. (Xiamen, China). The cell counting kit-8 (CCK-8) was obtained from Dojindo (Beijing, China). In addition, 4’, 6-diamino-2-phenylindole (DAPI) and paraformaldehyde (POM, pH = 7.4) were purchased from Solarbio (Beijing, China). All reagents were of analytical grade.

### 3.2. Preparation and Characterization of Collagen

Collagen was prepared by acid treatment according to the methods described by Li et al. [52] with slight modifications. Before preparation, the adhering residue tissues of skins were removed manually. Then, the non-collagenous proteins and pigments of skins were removed by treatment with 10 volumes of 0.1 mol/L NaHCO_3_ for 6 h. As shown in Figure 1, the pretreated skins were soaked in 0.5 M acetic acid with a sample-to-solvent ratio of 1:40 (*w*/*v*) for 24 h. The extracted liquid was then centrifuged at 9000× *g* for 30 min. The supernatant was precipitated by adding 4% NaCl, salted out for 30 min, and allowed to rest for 30 min. The resulting precipitate was collected using a freezing high-speed centrifuge (J-26 XP, Beckman Coulter Inc., Miami, FA, USA) at 9000× *g* for 30 min. The supernatant was then discarded, and the precipitate was removed until no precipitate remained. The collection was then dissolved and redispersed at a 1:9 (*w*/*v*) ratio in 0.5 M acetic acid and dialyzed against 20 volumes of 0.1 M acetic acid for 24 h, followed by 24 h of dialysis with distilled water five times. Thereafter, the tilapia skin collagen was lyophilized and stored at −20 °C until further use. All of these steps were conducted at temperatures below 4 °C.

The SDS-PAGE of the sample was conducted in accordance with the method of Chen et al. [53] with slight modifications. The samples were dissolved in cold distilled water and mixed at a 4:1 *v*/*v* ratio with sample loading buffer (277.8 mM Tris-HCl, pH 6.8, 44.4% (*v*/*v*) glycerol, 4.4% SDS, and 0.02% bromophenol blue) followed by boiling for 10 min. Next, 10 μL of samples were loaded onto a gel consisting of 8% separating gel and 3% stacking gel at a constant voltage of 110 V for electrophoresis (Bio-Rad Laboratories, Hercules, CA, USA). After electrophoresis for 90 min, the gel was soaked in 50% (*v*/*v*) methanol and 10% (*v*/*v*) acetic acid followed by staining with 0.125% CBB R-250 containing 50% (*v*/*v*) methanol and 10% (*v*/*v*) acetic acid. The gel was finally destained with a mixture of 50% (*v*/*v*) ethanol and 10% (*v*/*v*) acetic acid for 30 m. Marker 46634 was used to estimate the molecular weight of collagen, and type I collagen from rat tail was used as a standard.

### 3.3. Collagen-Based Composite Electrospun Fiber Membranes

The 4% collagen and 3% TPU were dissolved in HFIP separately in accordance with Jiang et al. [54]. A series of collagen-based composite spinning solutions were prepared with collagen and TPU solutions in different ratios (100:0, 95:5, 90:10, 80:20, 60:40) at room temperature. The spinning solutions were stirred using a magnetic stirrer (RCT digital S025, IKA, Staufen, Germany) until the solution was uniform and free of bubbles after mixing for 1 h. The mixture was then placed in a 2.5 mL syringe. The collagen-based composite electrospun fiber membranes were fabricated using an electrospinning apparatus (WL-2, Beijing Albizhi Ion Technology Co. Ltd., Beijing, China) with an applied voltage of 20 kV. The distance from the needle to the collector plate was 15 cm, and the propelling rate of the pump was 0.1 mL/h. The entire electrospinning process was conducted at room temperature at a relative humidity of 30–50%. The samples obtained from electrospinning were dried in a desiccator overnight to remove any residual organic solvent until use.

### 3.4. Structural Analysis of Col-TPU Nanofiber Membranes

#### 3.4.1. SEM

The morphology of the collagen-based composite electrospun fiber membranes was visualized using a scanning electron microscope (Quanta 450, FEI, Hillsboro, OR, USA). The sample was fixed on the sample platform with conductive adhesive, and sputtered with a gold coating for 30 s. The images were captured with SEM, with an accelerating voltage of 5–10 kV. The average nanofiber diameter of each sample was randomly measured using ImageJ (version 1.8.0, National Institute of Health, Bethesda, OR, USA) software in parallel three times by calculating the average and standard deviation per micrograph with more than 50 counts per image.

#### 3.4.2. FTIR

The infrared spectra of the samples were obtained with a Bruker FTIR spectrophotometer (Tensor27, Bruker, Madison, Germany) at room temperature. The samples were mixed with KBr by grinding at the ratio of 1:100 (*w*/*w*). The wavelength range was 4000-400 cm^−1^ with a resolution of 4 cm^−1^. The signals were collected automatically over 32 scans and ratioed against a background spectrum recorded from KBr. The secondary structure of the samples was then analyzed with OMNIC^TM^ software (Version 8.2, Thermo Nicolet Corporation, Madison, WI, USA) and PeakFit software (Version 4.12, Systat Software Inc., San Jose, CA, USA).

#### 3.4.3. XRD

The diffractograms of the samples were recorded with an X-ray diffractometer (X’Pert Pro XRD, PANalytical, The Netherlands) operating at 40 kV and 25 mA with CuKα radiation (λ = 1.5418 Å). The data were collected at a scanning speed of 10°·min^−1^ and a 2θ range of 5–90°. 

### 3.5. DSC

The DSC curves were obtained using a DSC instrument (DSC 204 F1, Netzsch, Selb, Bavaria, Germany) and the method reported by Kun et al. [55]. A certain amount of the samples (approximately 2–5 mg) were loaded into the bottom of an aluminum crucible and pressed with a lid. The samples were then transferred to the DSC instrument. During DSC scanning, the sample was cooled quickly with liquid nitrogen to −60 °C from room temperature and then heated to 200 °C at a heating rate of 10 °C/min in nitrogen atmosphere (purge flow of 50 mL/min and protective flow of 70 mL/min). Indium metal standard was used for temperature calibration.

### 3.6. Thermal Stability

The thermogravimetric analysis was conducted according to the methods described by Krishnakumar et al. [56] with some modifications. Thermogravimetric analysis (TGA2, Mettler Toledo Co. Ltd., Shanghai, China) used continuous nitrogen (flow rate 50 mL/min) in the sample chamber. Approximately 1 mg of the sample was placed in a crucible and pressed to create complete contact followed by sequential heating from 20 °C to 600 °C at a constant heating rate of 10 °C/min; the thermogravimetric data at the first heating curve were then recorded. 

### 3.7. WCA

The static water contact angle was recorded following the protocol reported by Lalia et al. [57]. The WCA of all samples was studied with a video optical contact angle measurement instrument (OCA15EC, DataPhysics, Filderstadt, Germany) and the sessile drop method. The samples were fixed on a glass slide, and deionized water (2 μL) was dispensed slowly onto the surface with a water rate of 5 μL/s. The water contact angle was measured at 3 s, and five different sites were measured from each sample to determine the uniform distribution of the samples. The images and calculation of the angle of the drop contact surface on both sides were recorded and analyzed with SCA20 (DataPhysics, Filderstadt, Germany) software.

### 3.8. Mechanical Properties

Sample mechanical properties were measured using a uniaxial tensile test according to the methods described by Zhu et al. [58]. Samples were trimmed into 1 cm × 5 cm strips, and both ends (1 cm on each side) of the samples were gripped by the fiber tensile tester (XQ−1C, New Fiber Instrument Co. Ltd., Shanghai, China). Material stress–strain curves were obtained through load deformation. The data were recorded by measuring the tensile strength and elongation at break at a tensile speed of 20 mm/min. 

### 3.9. Cytocompatibility

#### 3.9.1. Cell Proliferation

The proliferation evaluation of samples on MC3T3-E1 (Cat No. CBP60946) cells lines used a CCK-8 assay as reported by Yuan et al. [59] with slight modifications. The Col-TPU composite nanofiber membranes were spun on 14 mm-diameter round coverslips. Before culturing, samples were soaked in 75% ethanol for 30 min and UV-sterilized for 1 h. Subsequently, the samples were placed in a 24-well culture plate. After sterilization with 75% ethanol for 30 min, cells were rinsed three times with sterilized PBS solution (0.1 M) and cultured in Roswell Park Memorial Institute (RPMI)−1640 medium (Gibco, CA, USA) containing 10% fetal bovine serum (FBS) and 1% penicillin-streptomycin mixture (100 unit/mL penicillin, 100 μg/mL streptomycin) (Solarbio, Beijing, China). The cell density of MC3T3-E1 cells was adjusted to 1 × 10^4^ cells/well and seeded onto the samples cultured in the 24-well plate. The cells were cultured in a cell incubator with an atmosphere of 5% CO_2_ at 37 °C (HERAcell 150i, Thermo Scientific, Waltham, MA, USA). The CCK-8 solution was added to each well at 1, 2, and 3 days after culture. The optical densities of each well were measured using a multi-function microporous plate analyzer (Mithras2 LB 943, Berthold, Germany). The 14 mm-diameter round coverslips with no samples were used as the control; wells with no samples or coverslips were used as the blank; and the cell proliferation was calculated as follows:

Cell proliferation (%) = A_s_ − A_0_/A_c_ − A_0_ × 100%, (1)

where A_c_, A_s_, and A_b_ were the absorbance at 450 nm of the control group, the experimental group, and the blank group, respectively.

#### 3.9.2. Cell Morphology

Samples containing cultured MC3T3-E1 were immobilized in 2.5% POM for 3 days after inoculation. Before observation, the samples were washed with PBS and then washed with distilled water at least three times. Finally, all samples were gold-sputtered before cell morphologies were examined using SEM.

#### 3.9.3. Cell Adhesion

After 1, 2, and 3 days of cell inoculation, the MC3T3-E1 cells were fixed with 2.5% glutaraldehyde at pH = 7.4. During observation, the sample was washed with PBS three times for 5 min each, and the excess water was absorbed by the filter paper. After removal, the cells were inverted with 10 μL of DAPI and stained for 5 min. The adhesion of cells was observed with a laser scanning confocal microscope (LSCM) (TCSSP5, Leica Microsystems, Heerbrugg, Germany) or a positive fluorescence microscope (Axio Imager A2, ZEISS, Oberkochen, Germany). After 3 days of cell inoculation, the cells were fixed in 5% POM, and cell growth was observed using an automatic Cellomics Arrayscan (VTI-HCS, Thermo Scientific, Waltham, MA, USA).

### 3.10. Statistical Analyses

The analysis of variance was calculated using SPSS Version 17.0 software (IBM SPSS Statistics, Ehningen, Germany), and a value of *p* < 0.05 was used to indicate a significant deviation. Different letters indicate significant differences between samples.

## 4. Conclusions

In this study, collagen was extracted from tilapia fish skin and identified as type I collagen by SDS-PAGE. A series of Col-TPU composite nanofiber membranes (Col95, Col90, Col80, and Col60) were prepared via electrospinning and shown to be stable and have a nanostructure. There was relatively good compatibility between collagen and TPU. Besides maintaining the triple-helical structures of collagen, the addition of TPU enhanced the porosity, thermal stability, and mechanical properties of the composite. Thus, it was found to be more suitable for human tissue environments for long-term growth. In vitro fibroblast culture demonstrated a high cell proliferation rate with no cytotoxicity. The Col-TPU composite nanofiber membrane allowed the proliferation and migration of MC3T3-E1 cells and promoted fibrogenesis of cells; there was good biocompatibility. These results suggested that Col-TPU composite materials with different ratios of TPU were candidate biomaterials for tissue repair.

## Figures and Tables

**Table 1:** SEM results of Col-TPU composite nanofiber membranes.

Ratio (Col:TPU)	Abbreviation	Average Diameter (nm)	Porosity (%)
100:0	Col00	379.96 ± 134.28	46.59
95:5	Col95	378.40 ± 151.87	47.29
90:10	Col90	316.80 ± 94.51	48.81
80:20	Col80	313.80 ± 102.88	49.15
60:40	Col60	232.94 ± 87.82	52.89

**Table 2:** Properties of Col-TPU composite nanofiber membranes.

Samples	Thickness (mm)	Width (mm)	Breaking Strength (cN)	Tensile Strain (%)	Tensile Strength (MPa)
Col100	10.50	0.0242	36.21	3.20	1.40
Col95	9.80	0.0264	43.26	8.90	1.64
Col90	10.20	0.0200	44.06	16.50	2.12
Col80	10.60	0.0190	51.10	41.90	2.49
Col60	10.40	0.0174	56.28	48.30	3.05

## Data Availability

Data is contained within the article or Supplementary Materials.

## Supplementary Materials

The following supporting information can be downloaded at: https://www.mdpi.com/article/10.3390/md20070437/s1, Figure S1: HCA scan of Col-TPU composite nanofiber membranes, scale bar is 150 μm; Table S1: Comparison of Col-TPU composite nanofiber membranes different spectral peak positions in FTIR spectra; Table S2: The thermogravimetric analysis of Col-TPU composite nanofiber membranes; Table S3: CCK-8 assay OD_450_ of Col-TPU composite nanofiber membranes. The sequence of letters a–d represents the size of the mean value (a > b > c > d). The same letter indicates no statistically significant difference (*p* < 0.05, *n* = 4); Table S4: Cell proliferation and cytotoxicity evaluation of Col-TPU composite nanofiber membranes respect to control, % and grade.

## References

[1] Hart D.A., Nakamura N., Shrive N.G. (2021). Perspective: Challenges presented for regeneration of heterogeneous musculoskeletal tissues that normally develop in unique biomechanical environments. Front. Bioeng. Biotechnol..

[2] Shekhter A.B., Fayzullin A.L., Vukolova M.N., Rudenko T.G., Osipycheva V.D., Litvitsky P.F. (2019). Medical applications of collagen and collagen-based materials. Curr. Med. Chem..

[3] Mohammadalizadeh Z., Bahremandi-Toloue E., Karbasi S. (2022). Synthetic-based blended electrospun scaffolds in tissue engineering applications. J. Mater. Sci..

[4] Dorthe E.W., Williams A.B., Grogan S.P., D’Lima D.D. (2022). Pneumatospinning biomimetic scaffolds for meniscus tissue engineering. Front. Bioeng. Biotechnol..

[5] Law J.X., Liau L.L., Saim A., Yang Y., Idrus R. (2017). Electrospun collagen nanofibers and their applications in skin tissue engineering. Tissue Eng. Regener. Med..

[6] Acheson D., MacKnight C. (2019). Clinical implications of bovine spongiform encephalopathy. Clin. Infect. Dis..

[7] Wang L., Zou Y.F., Jiang S., Xu J.M., Jiang S.H., Hu Q.H. (2011). Chromatographic separation and physicochemical properties of collagen species in the skin of deep-sea redfish (*Sebastes mentella*). Food Hydrocoll..

[8] Chen J.D., Wang G.Y., Li Y.S. (2021). Preparation and characterization of thermally stable collagens from the scales of lizardfish (*Synodus macrops*). Mar. Drugs.

[9] Yunoki S., Hatayama H., Ohyabu Y., Kobayashi K. (2022). Fibril matrices created with collagen from the marine fish barramundi for use in conventional three-dimensional cell culture. Int. J. Biol. Macromol..

[10] Huang R.Q., Chen S.W., Ma G.Z., Yang B.W., Guo R.J., Li Q.X., Zhong J.Y. (2011). Research on the extraction of collagen from scales of tilapia. Adv. Mater. Res..

[11] Liu C.W., Hsieh C.Y., Chen J.Y. (2022). Investigations on the wound healing potential of tilapia piscidin (TP)2-5 and TP2-6. Mar. Drugs.

[12] Ge B.S., Wang H.N., Li J., Liu H.H., Yin Y.H., Zhang N.L., Qin S. (2020). Comprehensive assessment of nile tilapia skin (*Oreochromis niloticus*) collagen hydrogels for wound dressings. Mar. Drugs.

[13] Jin L., Zheng D.X., Yang G.Y., Li W., Yang H., Jiang Q., Chen Y.J., Zhang Y.X., Xie X. (2020). Tilapia skin peptides ameliorate diabetic nephropathy in STZ-induced diabetic rats and HG-induced GMCs by Improving mitochondrial dysfunction. Mar. Drugs.

[14] Jin S., Sun F., Zou Q., Huang J.H., Zuo Y., Li Y.B., Wang S.P., Cheng L., Man Y., Yang F. (2019). Fish collagen and hydroxyapatite reinforced poly(lactide-co-glycolide) fibrous membrane for guided bone regeneration. Biomacromolecules.

[15] He X.L., Wang L., Lv K.N., Li W.J., Qin S., Tang Z.H. (2022). Polyethylene oxide assisted fish collagen-poly-epsilon-caprolactone nanofiber membranes by electrospinning. Biomacromolecules.

[16] Zhou T., Sui B.Y., Mo X.M., Sun J. (2017). Multifunctional and biomimetic fish collagen/bioactive glass nanofibers: Fabrication, antibacterial activity and inducing skin regeneration in vitro and in vivo. Int. J. Nanomed..

[17] Xu Z.C., Wang X.Y., Huang H. (2020). Thermoplastic polyurethane–urea elastomers with superior mechanical and thermal properties prepared from alicyclic diisocyanate and diamine. J. Appl. Polym. Sci..

[18] Tatai L., Moore T.G., Adhikari R., Malherbe F., Jayasekara R., Griffiths I., Gunatillake P.A. (2007). Thermoplastic biodegradable polyurethanes: The effect of chain extender structure on properties and in-vitro degradation. Biomaterials.

[19] Enayati M., Eilenberg M., Grasl C., Riedl P., Kaun C., Messner B., Walter I., Liska R., Schima H., Wojta J. (2016). Biocompatibility assessment of a new biodegradable vascular graft via in vitro co-culture approaches and in vivo model. Ann. Biomed. Eng..

[20] Xu C.C., Hong Y. (2022). Rational design of biodegradable thermoplastic polyurethanes for tissue repair. Bioact. Mater..

[21] Lee J.K., Kang S.I., Kim Y.J., Kim M.J., Heu M.S., Choi B.D., Kim J.S. (2016). Comparison of collagen characteristics of sea- and freshwater-rainbow trout skin. Food Sci. Biotechnol..

[22] Gaspar-Pintiliescu A., Anton E.D., Iosageanu A., Berger D., Matei C., Mitran R.A., Negreanu-Pirjol T., Craciunescu O., Moldovan L. (2021). Enhanced wound healing activity of undenatured type I collagen isolated from discarded skin of black sea gilthead bream (*Sparus aurata*) conditioned as 3d porous dressing. Chem. Biodivers..

[23] Abbas A.A., Shakir K.A., Walsh M.K. (2022). Functional properties of collagen extracted from catfish (*Silurus triostegus*) waste. Foods.

[24] Sun L.C., Du H., Wen J.X., Zhong C., Liu G.M., Miao S., Cao M.J. (2021). Physicochemical properties of acid-soluble collagens from different tissues of large yellow croaker (*Larimichthys crocea*). Int. J. Food Sci. Technol..

[25] Fang H., Zhang L.J., Chen A.L., Wu F.J. (2022). Improvement of mechanical property for PLA/TPU blend by adding pla-tpu copolymers prepared *via* in situ ring-opening polymerization. Polymers.

[26] Džunuzović J.V., Stefanović I.S., Džunuzović E.S., Dapčević A., Šešlija S.I., Balanč B.D., Lama G.C. (2019). Polyurethane networks based on polycaprolactone and hyperbranched polyester: Structural, thermal and mechanical investigation. Prog. Org. Coat..

[27] Chen J.D., Li L., Yi R.Z., Xu N.H., Gao R., Hong B.H. (2016). Extraction and characterization of acid-soluble collagen from scales and skin of tilapia (*Oreochromis niloticus*). LWT-Food Sci. Technol..

[28] Jalan A., Kastner D.W., Webber K.G.I., Smith M.S., Price J.L., Castle S.L. (2017). Bulky dehydroamino acids enhance proteolytic stability and folding in beta-hairpin peptides. Org. Lett..

[29] Lin T.A., Lin J.-H., Bao L. (2021). A study of reusability assessment and thermal behaviors for thermoplastic composite materials after melting process: Polypropylene/thermoplastic polyurethane blends. J. Clean. Prod..

[30] Pei Y., Jordan K.E., Xiang N., Parker R.N., Mu X., Zhang L., Feng Z.B., Chen Y., Li C.M., Guo C.C. (2021). Liquid-exfoliated mesostructured collagen from the bovine achilles tendon as building blocks of collagen membranes. ACS Appl. Mater. Interfaces.

[31] Fang C.Q., Yang R., Zhang Z.S., Zhou X., Lei W.Q., Cheng Y.L., Zhang W., Wang D. (2018). Effect of multi-walled carbon nanotubes on the physical properties and crystallisation of recycled PET/TPU composites. RSC Adv..

[32] Liu J., Zhang L., Ci M.Y., Sui S.Y., Zhu P. (2020). Study on the rheological properties of regenerated cellulose/thermoplastic polyurethane blend spinning solutions. Ferroelectrics.

[33] Lopes M.S., Catelani T.A., Nascimento A.L., Garcia J.S., Trevisan M.G. (2019). Ketoconazole: Compatibility with pharmaceutical excipients using DSC and TG techniques. J. Therm. Anal. Calorim..

[34] Yehia A.A., Mansour A.A., Stoll B. (1997). Detection of compatibility of some rubber blends by DSC. J. Therm. Anal. Calorim..

[35] Frick A., Rochman A. (2004). Characterization of TPU-elastomers by thermal analysis (DSC). Polym. Test..

[36] Saha P., Khomlaem C., Aloui H., Kim B.S. (2021). Biodegradable polyurethanes based on castor oil and poly (3-hydroxybutyrate). Polymers.

[37] Bacakova L., Filova E., Parizek M., Ruml T., Svorcik V. (2011). Modulation of cell adhesion, proliferation and differentiation on materials designed for body implants. Biotechnol. Adv..

[38] Jing X., Li X., Jiang Y.F., Zhao R.H., Ding Q.J., Han W.J. (2021). Excellent coating of collagen fiber/chitosan-based materials that is water- and oil-resistant and fluorine-free. Carbohydr. Polym..

[39] Chen L., Yan C., Zheng Z. (2018). Functional polymer surfaces for controlling cell behaviors. Mater. Today.

[40] Cui Y., Yang Y., Qiu D. (2020). Design of selective cell migration biomaterials and their applications for tissue regeneration. J. Mater. Sci..

[41] Arhant M., Gall M.L., Gac P.-Y.L. (2022). Fracture test to accelerate the prediction of polymer embrittlement during aging-case of PET hydrolysis. Polym. Degrad. Stab..

[42] Hasan A., Memic A., Annabi N., Hossain M., Paul A., Dokmeci M.R., Dehghani F., Khademhosseini A. (2014). Electrospun scaffolds for tissue engineering of vascular grafts. Acta Biomater..

[43] Assanah F., Khan Y. (2018). Cell responses to physical forces, and how they inform the design of tissue-engineered constructs for bone repair: A review. J. Mater. Sci..

[44] Hasan A., Ragaert K., Swieszkowski W., Selimovic S., Paul A., Camci-Unal G., Mofrad M.R., Khademhosseini A. (2014). Biomechanical properties of native and tissue engineered heart valve constructs. J. Biomech..

[45] Richardson B.M., Walker C.J., Maples M.M., Randolph M.A., Bryant S.J., Anseth K.S. (2021). Mechanobiological interactions between dynamic compressive loading and viscoelasticity on chondrocytes in hydrazone covalent adaptable networks for cartilage tissue engineering. Adv. Healthc. Mater..

[46] Fang R., Zhang E.W., Xu L., Wei S.C. (2010). Electrospun PCL/PLA/HA based nanofibers as scaffold for osteoblast-like cells. J. Nanosci. Nanotechnol..

[47] Zheng X., Ke X., Yu P., Wang D.Q., Pan S.Y., Yang J.J., Ding C.M., Xiao S.M., Luo J., Li J.S. (2020). A facile strategy to construct silk fibroin based GTR membranes with appropriate mechanical performance and enhanced osteogenic capacity. J. Mater. Chem. B.

[48] Zhang Q., Lv S., Lu J.F., Jiang S.T., Lin L. (2015). Characterization of polycaprolactone/collagen fibrous scaffolds by electrospinning and their bioactivity. Int. J. Biol. Macromol..

[49] Liu H., Liu W.J., Luo B.H., Wen W., Liu M.X., Wang X.Y., Zhou C.R. (2016). Electrospun composite nanofiber membrane of poly(l-lactide) and surface grafted chitin whiskers: Fabrication, mechanical properties and cytocompatibility. Carbohydr. Polym..

[50] Zarei M., Samimi A., Khorram M., Abdi M.M., Golestaneh S.I. (2021). Fabrication and characterization of conductive polypyrrole/chitosan/collagen electrospun nanofiber scaffold for tissue engineering application. Int. J. Biol. Macromol..

[51] Bian T.R., Zhang H., Xing H.Y. (2020). Preparation and biological properties of collagen/nano-hydroxyapatite composite nanofibers based on ordered nano-hydroxyapatite ceramic fibers. Colloids Surf. A Physicochem. Eng. Asp..

[52] Li L.Y., Zhao Y.Q., He Y., Chi C.F., Wang B. (2018). Physicochemical and antioxidant properties of acid- and pepsin-soluble collagens from the scales of miiuy croaker (*Miichthys miiuy*). Mar. Drugs.

[53] Chen J.D., Li L., Yi R.Z., Gao R., He J.L. (2018). Release kinetics of tilapia scale collagen I peptides during tryptic hydrolysis. Food Hydrocoll..

[54] Jiang L., Jiang Y.C., Stiadle J., Wang X.F., Wang L.X., Li Q., Shen C.Y., Thibeault S.L., Turng L.S. (2019). Electrospun nanofibrous thermoplastic polyurethane/poly(Glycerol sebacate) hybrid scaffolds for vocal fold tissue engineering applications. Mater. Sci. Eng. C.

[55] Cong K., He J.Y., Yang R.J. (2021). Using twin screw extrusion reaction (TSER) to produce thermoplastic polyurethane (TPU): Tunable, stoichiometric and eco-friendly. Polym. Adv. Technol..

[56] Krishnakumar G.S., Gostynska N., Dapporto M., Campodoni E., Montesi M., Panseri S., Tampieri A., Kon E., Marcacci M., Sprio S. (2018). Evaluation of different crosslinking agents on hybrid biomimetic collagen-hydroxyapatite composites for regenerative medicine. Int. J. Biol. Macromol..

[57] Lalia B.S., Janajreh I., Hashaikeh R. (2017). A facile approach to fabricate superhydrophobic membranes with low contact angle hysteresis. J. Membr. Sci..

[58] Zhu Z.G., Wang W., Qi D.P., Luo Y.F., Liu Y.R., Xu Y., Cui F.Y., Wang C., Chen X.D. (2018). Calcinable polymer membrane with revivability for efficient oily-water remediation. Adv. Mater..

[59] Yuan M.Q., Dai F.F., Li D., Fan Y.Q., Xiang W., Tao F.H., Cheng Y.X., Deng H.B. (2020). Lysozyme/collagen multilayers layer-by-layer deposited nanofibers with enhanced biocompatibility and antibacterial activity. Mater. Sci. Eng. C.

