# Supplementary material for: Preparation and Characterization of Tilapia Collagen-Thermoplastic Polyurethane Composite Nanofiber Membranes
Source: Mar Drugs. 2022 Jun 30;20(7):437. doi: 10.3390/md20070437 (PMC9322160; doi:10.3390/md20070437)
Supplement: Supplementary file 1 [file marinedrugs-20-00437-s001.zip › marinedrugs-1769735-supplementary.pdf]

marine drugs

Supplementary Materials

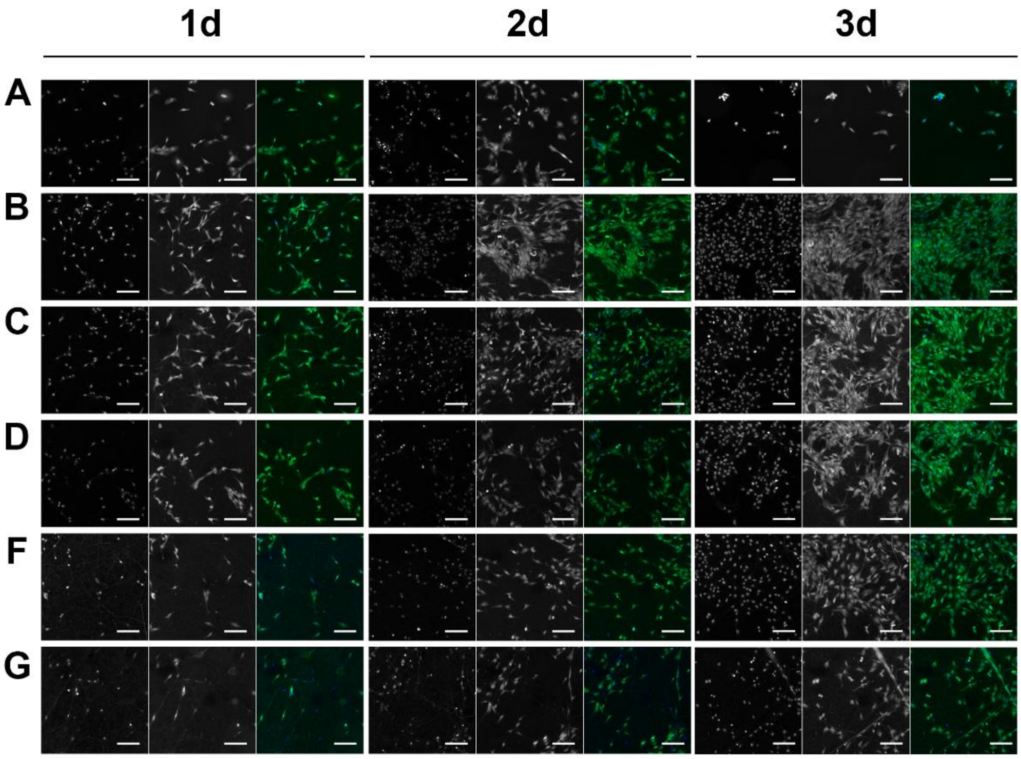

Figure S1. HCA scan of Col-TPU composite nanofiber membranes, scale bar is 150 μm.

Table S1. Comparison of Col-TPU composite nanofiber membranes different spectral peak positions in FTIR spectra.

Absorption peaks (cm^{-1})	Col100	Col95	Col90	Col80	Col60	TPU
Around 3315 (Amide A)	3311	3307	3307	3307	3309	-
N-H (TPU)	-	3306↑	3307↑	3307↑	3308↑	3330
2920-2944 (Amide B)	2932	2932↑	2933↑	2936↑	2939↑	-
C-H (TPU)	-	-	-	-	-	2942
1625-1690 (Amide I)	1655	1655	1654	1654	1654	-
C=C (TPU)	-	1538↓	1537↓	1535↓	1534↓	1532
1500-1600 (Amide II)	1538	1538	1537	1535	1534	-
CH_2 (TPU)	-	1452↓	1451↓	1450↓	1449↓	1447
1200-1300 (Amide III)	1223	1226	1231	1228	1227	-
-NHCOO-	-	-	-	-	-	1240
C-O-C (TPU)	-	1000↑	1101↑	1107↑	1108↑	1100

Table S2. The thermogravimetric analysis of Col-TPU composite nanofiber membranes.

Samples	$T_{5\%}$, °C	Tp, °C	Tp weight loss, %	$T_{50\%}$, °C	Decomposition of TPU, °C	Residue at 600 °C
Col100	65.0	314.0	54.81	327.0	-	14.39
Col95	57.7	319.0	56.29	331.0	400.3	15.34
Col90	64.3	320.5	62.26	343.6	413.8	16.32
Col80	68.3	320.7	62.70	350.3	419.7	18.57
Col60	75.7	321.8	65.68	357.0	421.5	19.67

Table S3. CCK-8 assay OD_{450} of Col-TPU composite nanofiber membranes. The sequence of letters a-d represents the size of the mean value (a>b>c>d). The same letter indicates no statistically significant difference ($P<0.05$, $n=4$).

Samples	1d	2d	3d
Control	0.18^{abc}	0.36^{b}	0.56^{c}
Col100	0.19^{a}	0.78^{a}	1.76^{a}
Col95	0.18^{ab}	0.40^{b}	1.11^{b}
Col90	0.17^{abc}	0.35^{b}	1.00^{b}
Col80	0.15^{cd}	0.34^{b}	0.97^{b}
Col60	0.17^{d}	0.32^{b}	0.93^{b}

Table S4. Cell proliferation and cytotoxicity evaluation of Col-TPU composite nanofiber membranes respect to control,% and grade.

Samples	1d		2d		3d	
	%	grade	%	grade	%	grade
Col100	107.04	0	215.53	0	312.55	0
Col95	102.25	0	110.40	0	195.93	0
Col90	97.04	0	97.43	0	177.86	0
Col80	81.97	I	94.24	0	171.66	0
Col60	76.80	I	88.21	0	164.92	0
